# Curcumin Sensitizes Kidney Cancer Cells to TRAIL-Induced Apoptosis via ROS Mediated Activation of JNK-CHOP Pathway and Upregulation of DR4

**DOI:** 10.3390/biology9050092

**Published:** 2020-05-01

**Authors:** Ismael Obaidi, Hilary Cassidy, Verónica Ibáñez Gaspar, Jasmin McCaul, Michael Higgins, Melinda Halász, Alison L. Reynolds, Breandan N. Kennedy, Tara McMorrow

**Affiliations:** 1 NIBRT|National Institute for Bioprocessing, Research and Training, Foster Avenue, Mount Merrion, Blackrock, Co., A94 X099 Dublin, Ireland; 2 UCD Centre for Toxicology, School of Biomedical and Biomolecular Sciences, Conway Institute, University College Dublin, 4 Dublin, Ireland; hilary.cassidy@ucd.ie (H.C.); veronica.ibanezgaspar@ucdconnect.ie (V.I.G.); jasmin.mc-caul@ucdconnect.ie (J.M.); michael.higgins.2@ucdconnect.ie (M.H.); alison.reynolds@ucd.ie (A.L.R.); brendan.kennedy@ucd.ie (B.N.K.); 3 College of Pharmacy, University of Babylon, Babylon 51002, Iraq; 4 Systems Biology Ireland, School of Medicine, University College Dublin, Belfield, 4 Dublin, Ireland; melinda.halasz@ucd.ie; 5 UCD School of Veterinary Medicine, Rm 232, University College Dublin, Belfield, 4 Dublin, Ireland

**Keywords:** TRAIL, curcumin, chemosensitisation, apoptosis, zebrafish, ROS, MAPK, DR4

## Abstract

Tumour necrosis factor-related apoptosis-inducing ligand (TRAIL), is a selective anticancer cytokine capable of exerting a targeted therapy approach. Disappointingly, recent research has highlighted the development of TRAIL resistance in cancer cells, thus minimising its usefulness in clinical settings. However, several recent studies have demonstrated that cancer cells can be sensitised to TRAIL through the employment of a combinatorial approach, utilizing TRAIL in conjunction with other natural or synthetic anticancer agents. In the present study, the chemo-sensitising effect of curcumin on TRAIL-induced apoptosis in renal carcinoma cells (RCC) was investigated. The results indicate that exposure of kidney cancer ACHN cells to curcumin sensitised the cells to TRAIL, with the combination treatment of TRAIL and curcumin synergistically targeting the cancer cells without affecting the normal renal proximal tubular epithelial cells (RPTEC/TERT1) cells. Furthermore, this combination treatment was shown to induce caspase-dependent apoptosis, inhibition of the proteasome, induction of ROS, upregulation of death receptor 4 (DR4), alterations in mitogen-activated protein kinase (MAPK) signalling and induction of endoplasmic reticulum stress. An in vivo zebrafish embryo study demonstrated the effectiveness of the combinatorial regime to inhibit tumour formation without affecting zebrafish embryo viability or development. Overall, the results arising from this study demonstrate that curcumin has the ability to sensitise TRAIL-resistant ACHN cells to TRAIL-induced apoptosis.

## 1. Introduction

Renal cancer is the ninth most common cancer in men and the 14th most incident cancer in women worldwide, with 403,262 newly diagnosed renal cancers cases and 175,098 deaths globally in 2018 [1]. In 2019, 1,762,450 new kidney cancer cases and 606,880 deaths were projected to occur in the United States [2]. Renal cell carcinoma (RCC) is the most common type of kidney cancer in adults, responsible for approximately 90–95% of cases. Due to lack of early-warning signs, complex clinical manifestations and resistance to chemotherapy, the advanced stages of RCC carry a particularly poor prognosis with only a 5% overall five-year survival rate [3].

Targeted therapies are frequently making their way from the bench into clinical practice because of their observed success in specifically targeting cancerous cells. The tumour necrosis factor-related apoptosis-inducing ligand (TRAIL) was heralded as a promising candidate for use clinically as a targeted therapeutic with research demonstrating that the agent had the ability to effectively inhibit different tumour types while displaying minimal toxicity on normal healthy cells. TRAIL is a trimeric ligand which binds to DR4 (TRAIL-R1) and/or DR5 (TRAIL-R2), activating the death inducing signalling cascade (DISC) which incorporates Fas associated death domain (FADD). FADD recruits and activates the initiator procaspase 8 to active caspase 8, which in turn amplifies the death signal by cleaving and activating other caspases including procaspase 3. Active caspase 3 is referred to as the executioner caspase since it induces the digestion of cellular cytoplasmic and nuclear proteins [4,5].

However, the development of TRAIL resistance by many cancer cells has substantially limited any potential therapeutic applications in a clinical setting [6]. Recent research into TRAIL resistance has shown that combinatorial regimes with other drugs, such as bortezomib and other proteasome inhibitors can reduce TRAIL-resistance [7]. Disappointingly, bortezomib has demonstrated high toxicity, thereby necessitating the continuation of the search for alternative treatments possessing the TRAIL-sensitising activity coupled with much lower toxicity [8,9,10].

**Figure 1:**
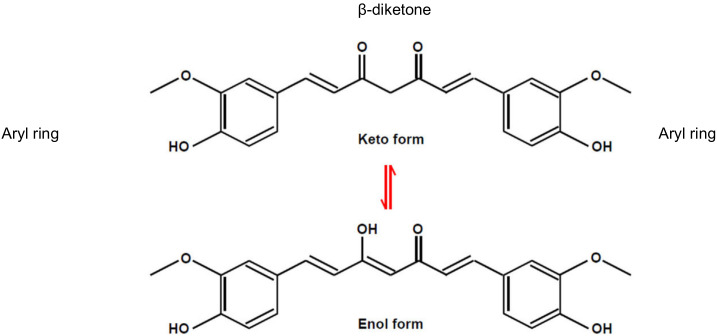
Chemical structure of curcumin [18].

Curcumin, a polyphenolic phytochemical which is the primary bioactive component isolated from the plant species curcuma longa, has demonstrated anti-proliferative and anti-carcinogenic properties with a very low toxicity profile, making it an attractive agent for cancer treatment [11,12,13,14,15,16,17]. Structurally, curcumin is one of the polyphenolic compounds that exhibits a characteristic keto-enol tautomerism structure (Figure 1) [18]. The β-diketone moiety plays an integral role in conjunction the two aryl moieties and impart the tautomeric structures of curcumin that affects photochemical and photophysical properties of the compound [19].

In this study, the chemosensitising potential of curcumin on TRAIL-induced apoptosis was investigated in vitro. Additionally, the tumour inhibitory effect of the curcumin and TRAIL combination treatment was assessed in a novel in vivo zebrafish model of RCC.

## 2. Materials and Methods

### 2.1. Cell Culture

The human renal proximal tubular epithelial cells (RPTEC/TERT1) and the renal carcinoma ACHN cells were obtained from the American Type Culture Collection (Manassas, VA, USA). RPTEC/TERT1 cells were maintained in low glucose (5 mM) Dulbecco’s Modified Eagle Medium/Nutrient Mix F-12 supplemented with 5 µg/mL insulin, 5 µg/mL transferrin, 5 µg/mL selenite (ITS), 36 ng/mL hydrocortisone, 10 ng/mL epidermal growth factor (EGF) (Sigma-Aldrich, St. Louis, MO, USA), 50 U/mL penicillin, 50 µg/mL streptomycin and 2 mM L-glutamine (Gibco, Life Technologies, Carlsbad, CA, USA). ACHN cells were grown in Minimum Essential Medium (Sigma-Aldrich, St. Louis, MO, USA) with 5% foetal bovine serum (FBS) (Gibco, Life Technologies, Carlsbad, CA, USA) and 50 U/mL penicillin and 50 µg/mL streptomycin.

For all experiments, 50 mM stock curcumin (Sigma-Aldrich, St. Louis, MO, USA) solution was prepared in DMSO (Sigma-Aldrich, St. Louis, MO, USA), then the working solution was prepared in culture medium at a dilution of 1:2000 (*v*/*v*) with the final DMSO concentration of 0.05%. TRAIL (Pepprotech, NJ, USA) powder was reconstituted (according to the manufacturer instruction) in sterile distilled water at a concentration of 500 µg/mL. Culture medium was used to prepare the final working concentration.

### 2.2. Cell Viability Assays

Different concentrations and combinations of curcumin and TRAIL were assessed. ACHN cells were seeded at a density of 7.5 × 105 cells/mL in 96-well plates. The next day, ACHN cells were exposed to culture medium containing DMSO or 5, 15, and 25 µM curcumin for 4 h, followed by further treatment with TRAIL (0, 10, 50 and 200 ng/mL) For a further 20 h (24 h exposure in total). RPTEC/TERT1 cells were treated in a similar fashion, however the RPTEC/TERT1 cells were seeded and maintained for 10 days post confluency to allow cells to fully differentiate. A highly sensitive resazurin-based assay called FluoroFire-BlueProViaTox (Molecutool, MSC, Ireland) was employed to assess cell viability. The fluorescent were measured at excitation 450 nM and emission of 590 nM using SpectraMax M2 plate reader (Molecular device, Sunnyvale, CA, USA).

### 2.3. Combination Index Analysis of Synergy

Combination index (CI) analysis was performed to determine, based on the median effect, whether the combination of curcumin with TRAIL had a synergistic anti-cancer activity. The software CalcuSyn (Biosoft, Ferguson, MO, USA) was used to calculate the combination index (CI) which determines the type of the interaction between the chemicals. CI = 1 indicates an additive effect, CI > 1 indicates antagonism, if CI < 1 means the interaction is synergistic. Another scoring system was recommended to indicate the degree of synergy [20]. In this context, +++, ++ and + indicate strong synergy, medium synergy, and moderate synergy, respectively.

### 2.4. Phase Contrast Microscopy

Cellular morphology was assessed by phase contrast microscopy using a JVC high-resolution digital camera (KY-F55BE) (JVC, Yokahama, Tokyo, Japan) attached to a Nikon TMS phase contrast microscope (Nikon, Shinagawa, Tokyo, Japan). Micrographs were processed using the ImageJ 1.49m program.

### 2.5. Flow Cytometry

For apoptosis and ROS assays, ACHN cells were cultured on six-well plates at a density of 5 × 10^5^ cells/mL. The next day, cells were incubated with culture medium containing 0.05% DMSO or 25 µM curcumin for 4 h prior to analysis. In parallel, ACHN cells were also incubated with culture medium containing 0.05% DMSO or 25M curcumin for 4 h followed by a further incubation with 50 ng/mL TRAIL as indicated. YO-PRO/PI kit (Thermo Fischer Scientific, Waltham, MA, USA) was used (according to the manufacturer instruction) to determine whether the treatments induced apoptotic or necrotic cell death. In brief, following incubation with the treatments, cells were detached, re-suspended and incubated with 1 µL YO-PRO-1 (100 µM in DMSO) for 15 min on ice, followed by adding 1 µL propidium iodide (PI) just before the analysis. For ROS detection, cells were incubated with 80 μM Carboxy-H2DFFDA (Thermo Fischer Scientific, Waltham, MA, USA) for 30 min at 37 °C. For the assessment of surface expression of death receptors, cells were treated with 0.05% DMSO or 25 µM curcumin for 4 h, followed by adding TRAIL to the final concentration of 50 ng/mL for up to 24 h. Cells were then harvested and incubated for 30 min at 4 °C with allophycocyanine (APC) or phycoerythrin (PE) conjugated monoclonal anti-human DR4 and DR5 antibody (BioLegend, San Diego, CA, USA), respectively. Depending on the lasers, for apoptosis assay, A CyAn ADP flow cytometer (Beckman Coulter, IN, USA) was employed in the analysis. For ROS and the surface expression of DRs, Accuri C6 flow cytometry (BD Biosciences, San Jose, CA, USA) was used. The resulting data was analyzed using FCS Express 6 software (Glendale, CA, USA).

### 2.6. Caspase Activity Assays

The activity of three of main caspases; caspase 8, -9, and -3/7 were quantified using specific fluorogenic AC-LETD-AFC, AC-LEHD-AFC and AC-DEVD-AFC substrates (ENZO, Exeter, UK) specific for these caspases, respectively. These substrates were incubated with caspase assay lysis buffer (CALB) that consists of 2.5 mL 50% (*w*/*v*) glycerol, 125 mg CHAPS, 125 mg 100 mM EDTA, 12.5 µL PMSF, 10.5 mg 50 mM DTT and 22.1 mL 1× PBS (all Sigma-Aldrich, St. Louis, MO, USA). For the assays, cells were seeded on six-well plates and the following day were treated with 25 µM curcumin for 4 h, followed by 50 ng/mL of TRAIL for 2, 4, 6, 8 and 20 h (total exposure times of 6, 8, 10, 12 and 24 h). Cells were harvested and centrifuged at 200× *g* at 4 °C for 8 min. Following two washes with 1× ice cold PBS, the cells were lysed in 100 µL CALB then 80 µL of each sample was loaded into a black wall 96-well plate. The fluorogenic substrate (100 mM stock) was diluted 1:1000 in CALB and 80 µL was added to the samples. CALB alone was employed as the negative control. The assay was kinetically performed at 37 °C for a 120 min period (120 cycle consisting of one measurement per minute), at an emission and excitation wavelength of 400 and 505 nm respectively. For the data analysis, the caspase activity was normalized against samples protein concentration.

### 2.7. Proteasome Assay

ACHN cells were cultured on six-well plates incubated with culture medium or 25 µM curcumin for 4 h. Following this, the cells were further incubated with the culture medium or 50 ng/mL TRAIL. Cells were lysed in proteasome lysis buffer (50 mM HEPES pH 7.8, 10 mM NaCl, 1.5 mM MgCl_2_, 1 mM EDTA, 0.2% Triton-X100, 250 mM sucrose, DTT 5 mM (all reagents supplied by Sigma-Aldrich, St. Louis, MO, USA). The lysates were transferred into ice pre-chilled Eppendorf tubes then sonicated for 10 s using microtip set on ~2. Following centrifugation (10,000× *g*) at 4 °C for 10 min, the supernatant was incubated with 100 μM N-succinyl-Leu-Leu-Val-tyr-7-amino-4-methyl-coumarin (SUC-LLVY-AMC) (ENZO, Exeter, UK) fluorogenic substrates in assay buffer (50 mM HEPES pH 7.8, 10 mM NaCl, 1.5 mM MgCl_2_, 1 mM EDTA, 250 mM sucrose and DTT 5 mM). The ATP (Sigma-Aldrich, St. Louis, MO, USA) solution was added to the proteasome assay buffer to get 2 mM final concentration just before adding the substrate. The release of the 7-amino-4-methyl-coumarin (AMC) was fluorometrically measured at an excitation/emission of 360/475 nm. The assay was kinetically measured for 60 min (60 cycles, one measurement per min) at 37 °C. To ensure that the observed activity was indeed proteasome derived, 10 μM MG-132 was added to the vehicle treated cell lysate that served as a positive control. The results were normalized for protein content using BCA protein assay (Sigma-Aldrich, St. Louis, MO, USA).

ACHN cells were cultured on six-well plates incubated with culture medium or 25 µM curcumin for 4 h. Following this, the cells were further incubated with the culture medium or 50 ng/mL TRAIL. Cells were lysed in proteasome lysis buffer (50 mM HEPES pH 7.8, 10 mM NaCl, 1.5 mM MgCl_2_, 1 mM EDTA, 0.2% Triton-X100, 250 mM sucrose, DTT 5 mM (all reagents supplied by Sigma-Aldrich, St. Louis, MO, USA). The lysates were transferred into ice pre-chilled Eppendorf tubes then sonicated for 10 s using microtip set on ~2. Following centrifugation (10,000× *g*) at 4 °C for 10 min, the supernatant was incubated with 100 μM N-succinyl-Leu-Leu-Val-tyr-7-amino-4-methyl-coumarin (SUC-LLVY-AMC) (ENZO, Exeter, UK) fluorogenic substrates in assay buffer (50 mM HEPES pH 7.8, 10 mM NaCl, 1.5 mM MgCl_2_, 1 mM EDTA, 250 mM sucrose and DTT 5 mM). The ATP (Sigma-Aldrich, St. Louis, MO, USA) solution was added to the proteasome assay buffer to get 2 mM final concentration just before adding the substrate. The release of the 7-amino-4-methyl-coumarin (AMC) was fluorometrically measured at an excitation/emission of 360/475 nm. The assay was kinetically measured for 60 min (60 cycles, one measurement per min) at 37 °C. To ensure that the observed activity was indeed proteasome derived, 10 μM MG-132 was added to the vehicle treated cell lysate that served as a positive control. The results were normalized for protein content using BCA protein assay (Sigma-Aldrich, St. Louis, MO, USA).

### 2.8. RNA Extraction, cDNA Extraction and qRT-PCR

ACHN cells were treated with either 25 µM curcumin, 50 ng/mL TRAIL or the combination of both agents for 24 h. The media was removed, and total RNA was extracted by the TRIzol method [21]. Briefly, cells were lysed in TRIzol (Ambion, Life Technologies, Carlsbad, CA, USA). The phase separation of lysates was induced by the addition of 1-bromo-3-chloropropane (BCP) into three phases. The upper aqueous layer (contains RNA) was removed and RNA was precipitated by adding isopropanol. RNA pellets were washed with ethanol then dissolved in a sufficient volume of RNase free water. For further RNA purification and removing of genomic DNA contamination, mRNeasy kit (Qiagen, Lloyd St N, Manchester UK) was used according to the manufacturer protocol. The concentration and purity of RNA samples were assessed by measuring the optical density at 260 nm and 280 nm using a Nanodrop ND-1000 spectrophotometer (Thermo Fischer Scientific, Waltham, MA, USA) following the manufacturer protocol. cDNA was synthesized using a RevertAid H Minus First Strand cDNA synthesis Kit (Fermentas GmbH, St. Leon-Rot, Germany) according to the manufacturer’s instructions. For qRT-PCR experiments, A TaqMan probe-based gene expression assay (Applied Biosystems, Foster City, CA, USA) was employed to measure DR4 gene expression. Briefly, the PCR reaction solution (10 μL) contained 0.5 μL cDNA, 3.5 μL nuclease free water, 0.5 μL forward and reverse primer mix, 0.5 μL forward and reverse loading control probe and 5 μL TaqMan master mix. The thermal cycle conditions were as follows: 50 °C for 2 min (activation of AMPerase), 95 °C for 10 min (Taq activation), followed by 40 cycles of 95 °C for 15 s for denaturation and 60 °C for 1 min for annealing and extension. Samples were loaded in an optical 384 well plate in duplicates (10 µL/well) and Ct values < 35 were used for analysis. The mRNA abundance of the target gene was normalized against the expression of GAPDH (Applied Biosystems, Foster City, CA, USA) loading control. The 2−ΔΔCt method was employed to analyse the results [22,23].

### 2.9. Western blot Analysis

Western blot analysis was performed according to the standard method described by Buchmann [24]. Following treatment, the media was removed, and the cells were lysed by in RIPA buffer (Sigma-Aldrich, St. Louis, MO, USA). Total protein concentration was determined using a BCA protein assay kit according to the manufacturer protocol (Pierce, Rockford, IL, USA). Samples were mixed with 3× blue sample buffer containing 50 mM dithiothreitol (Sigma-Aldrich, St. Louis, MO, USA) (reducing agent) then boiled at 100 °C for 3–5 min to denature proteins. An equivalent volume to 20 µg of the whole cell lysates were loaded into each lane and subjected to SDS-PAGE electrophoresis, then transferred to a 0.2 µm pore size nitrocellulose membrane Whatman Protran^®^ (Thermo Fisher Scientific, MA, USA) using a SemiPhor semi-dry transfer system (G. E. Healthcare, Chicago, IL, USA). The membranes were blocked by incubation in TBS-T buffer (50 mM tris-HCl, pH 7.4, 150 mM NaCl, and 0.05% Tween 20) containing 5% non-fat milk (Sigma-Aldrich, St. Louis, MO, USA) or BSA (Sigma-Aldrich, St. Louis, MO, USA) for 1 h at room temperature. The membranes were then incubated overnight at 4 °C with a primary antibody against either DR4 (1:250), Cdk1, Bcl-2 and p-P38 (Santa Cruz, Dallas, Texas, USA; all 1:1000 dilution), cFLIP (Enzo, Exeter, UK; 1:500 dilution), FADD (Enzo, Exeter, UK; all 1:1000 dilution), CHOP (Cell Signalling Technology Inc., MA, USA; 1:500 dilution). Bax, JNK, P-JNK, P38, p-P38, ERK, p-ERK, and GAPDH (Cell Signalling Technology Inc., MA, USA; all 1:1000 dilution). The blots were washed with TBS-T and incubated with the appropriate species of HRP coupled secondary antibody diluted in TBS-T containing 5% BSA or non-fat milk (Cell Signalling Technology Inc., Danvers, MA, USA; 1:2000 dilution) for 1 h at room temperature. Immunodetection was performed using the SuperSignal West Pico Substrate (Thermo Scientific, Rockford, IL, USA). Following the incubation of the blots with the Chemiluminescent Substrate, blots were air-dried and transferred to a sealed cassette and exposed to autoradiographic developing film for various lengths of time depending on the strength of the signal. The film was developed in an automatic developer. Following detection, the blots were washed three times with TBS-T, and re-probed for GAPDH loading control. If the bands located within GAPDH detection range, stripping buffer was used to strip off the blots before re-probing with GAPDH primary antibody. In some cases, samples from different timepoints were run on different blots due to logistical limitations, however, all samples were normalized to their GAPDH loading controls.

ImageJ software 1.50i (National Institutes of Health, Bethesda, MD, USA) (http://imagej.nih.gov/ij) was used for greyscaling and performing densitometry analysis of the bands. All presented blots were subjected to automatic corrections for brightness.

### 2.10. Zebrafish Toxicity Testing and Xenotransplantation

The wild type zebrafish (WT) were maintained according to the standard protocol by [25]. They were kept on an 10h/14 h artificial dark/light cycle at a temperature of 26 ± 1 °C. Following 48 h post fertilization, at least 5 fertilized larvae were placed in each well of 48-well culture plate in duplicates for toxicity testing. Embryos were treated with embryonic medium containing 25 µM curcumin, 50 ng/mL TRAIL or the combination of both for 72 h. Embryos exposed to embryonic medium containing 0.05% DMSO only were employed as a negative control. Following the incubation, zebrafish embryos were anesthetized with 0.002% tricaine (Sigma-Aldrich, St. Louis, MO, USA) then placed on a glass slide and inspected under the microscope for any abnormalities using Nikon SMZ645 (Tokyo, Japan).

The micro injector PV 830 Pneumatic Pico Pump (World Precision Parameter, Sarasota, FL, USA) was used to engraft ACHN cells into the yolk sac of zebrafish embryos. The approximate injection parameters were injection pressure = 100 kPa, holding pressure = 50 kPa and injection time = 0.8 s. Zebrafish embryos of 24-hpf were treated with 200 µM N-phenylthiourea (Alfa Aesar, UK) to prevent formation of pigments and to keep the embryos transparent. After 2 h of injection, embryos were assessed under a fluorescent microscope and those showing metastasis of cells were excluded. The injected embryos were kept at 33 °C for 72 h post injection for imaging using Olympus SZX10 fluorescent microscope with Burner- OLYMPUS U-RFL-T (Olympus SZX10 microscope, Hamburg, Germany). The images then were processed, and the relative fluorescent intensity was quantified by ImageJ software 1.50i (http://imagej.nih.gov/ij) was used for greyscaling and performing densitometry analysis of the bands. All presented blots were subjected to automatic corrections for brightness.

All in vivo experiments were carried out with approval granted by the UCD ethics committee (AREC-E-16-19-McMorrow) and conducted according to EU directive 2010/63/EU on the protection of animals used for scientific purposes.

### 2.11. Statistical Analysis

All experiments were analysed using GraphPad prism 5.0 (Graph Pad software, San Diego, CA, USA). Data were analysed, using ANOVA (one- and two-way) and the Bonferroni post-test was used to compare between different treatments groups. Alternatively, where appropriate, the unpaired student’s independent t-test was employed to test the statistical difference. Results were expressed as the mean ± standard error of the mean (SEM). A probability of 0.05 or less was considered as statistically significant. The software Image J was used for greyscaling and performing densitometry analysis of the Western blot bands.

## 3. Results

### 3.1. Combination Treatment of Curcumin and TRAIL Increases Cell Death in the Cancerous Renal ACHN Cells without Affecting the Normal Epithelial RPTEC/TERT1 Cells

**Figure 2:**
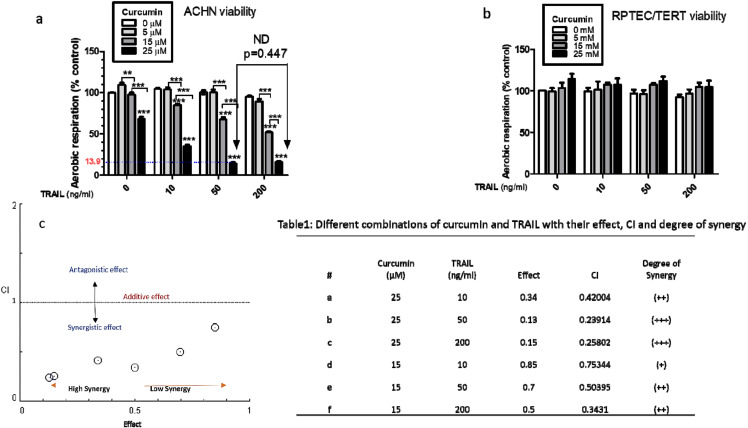
Effects of curcumin on cellular viability of cancerous ACHN and normal epithelial RPTEC/TERT1 cells. Cell viability was assessed in both (**a**) cancerous ACHN cells cultured for 24 h before treatment and (**b**) RPTEC/TERT1 cells cultured and treated 10 days post-confluency. Both cell lines were treated with curcumin (0, 5, 15 or 25 µM) for 4 h before exposure to TRAIL (0, 10, 50 or 200 ng/mL) until 24 h. Cell viability was assessed using the FluoroFire-Blue ProViaTox Resazurin Fluorescent assay, with the results expressed as the mean resorufin fluorescence (% of control) ± SEM of three independent experiments. ** and *** indicate statistically significant difference at *p* < 0.01 and *p* < 0.001, respectively. Two-way ANOVA was used to analyse the data (**c**) combination index blot and data show the interactions between TRAIL and curcumin based on medium or 50% effect level. The line indicates an additive effect, whereas values below are synergistic and the above are antagonistic. The degree of synergy is determined based on the calculated effect by CompuSyn software; less than 0.5 determined to have a higher degree of synergy while low synergy can be observed above than 0.5. Accordingly, a very strong synergistic interaction can be noticed between 25 µM curcumin with 200 or 50 ng/mL TRAIL.

The ability of curcumin to sensitise human renal cancerous ACHN cells to the apoptotic effects of TRAIL was investigated. It has previously been reported that ACHN cells are TRAIL resistant and thus were a suitable model for this study [26]. Two cell lines, both the cancerous renal ACHN and the normal renal epithelial RPTEC/TERT1, were treated with different concentrations of curcumin with/without TRAIL. The results demonstrated that the combination of 25 µM curcumin with 50 ng/mL TRAIL markedly enhanced the death of ACHN cells to approximately 90% compared to untreated cancerous cells (Figure 2a). Conversely, no cell death was observed in the RPTEC/TERT1 cells following the exposure to any of the treatments (Figure 2b). A combination index was utilized to show the synergistic combination of curcumin with TRAIL, with the highest synergism evident at the 25 µM curcumin with 50 or 200 ng/mL TRAIL dose (Figure 2c). Based on these findings the 25 µM curcumin with 50 ng/mL TRAIL was employed for all further studies.

### 3.2. Morphological Comparison between RPTEC/TERT1 and ACHN Cells upon Exposure to Curcumin, TRAIL and Curcumin/TRAIL Co-Treatment

**Figure 3:**
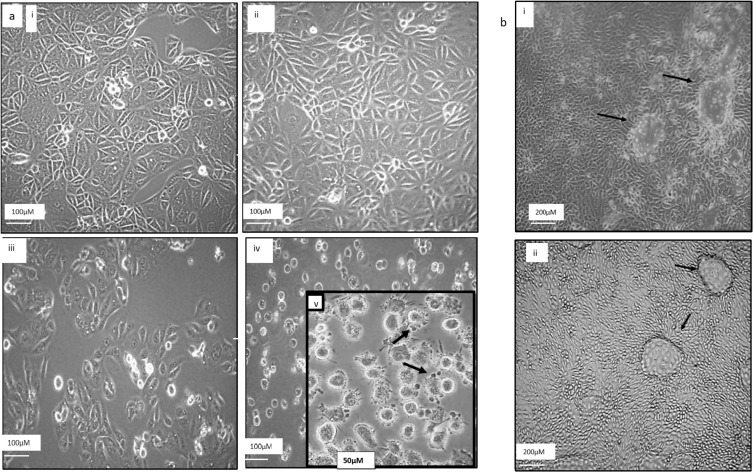
Effects of curcumin and TRAIL on the cell morphology (**a**) ACHN cells were treated with (**i**) culture medium contains 0.05% DMSO, (**ii**) 50 ng/mL TRAIL, (**iii**) 25 µM curcumin or (**iv**) 50 ng/mL TRAIL plus 25 μM Curcumin. ACHN cellular morphology was assessed using phase contrast microscopy under 100× magnification (scale 100 µM). (**v**) Higher magnification (identified bleb-like protrusions or “apoptotic bodies” (scale 50 µM). (**b**) RPTEC/TERT1 cells were cultured on six-well plates for 10 days to allow differentiation before treatment with (**i**) culture medium contains 0.05% DMSO only or with (**ii**) 50 ng/mL TRAIL plus 25 μM curcumin. The effects on cellular morphology were assessed. The arrows indicate the presence of fluid-filled dooms which reflect the establishment of an intact and functioning transport system within the cells and acts as an indicator of the overall RPTEC/TERT1 health (scale 200 µM).

Cells exposed to 0.05% DMSO or TRAIL showed a similar morphological pattern (Figure 3a i,ii). In contrast, curcumin was observed to adversely affect the cells as shown by low cell density, appearance of prominent nuclei, and cell shrinkage (Figure 3a iii), while the combination of 25 µM curcumin with 50 ng/mL TRAIL massively induced cell death as evident by the increase in the numbers of dead and floating cells (Figure 3a iv). At the higher magnification (400×), the typical phenotypic markers of apoptosis including cell shrinkage, nuclear condensation, cellular blebbing and formation of apoptotic bodies, all of which was evident in the ACHN cells following the combination treatment (Figure 3a v). In contrast, no changes in morphology were detected in the RPTEC/TERT1 cell line upon comparison of the vehicle control treated cells (Figure 3b i) and the 50 ng curcumin/25 μM TRAIL combination treatment (Figure 3b ii).

### 3.3. Curcumin Alone or in a Combination with TRAIL Induced Apoptosis in Renal ACHN Cells

**Figure 4:**
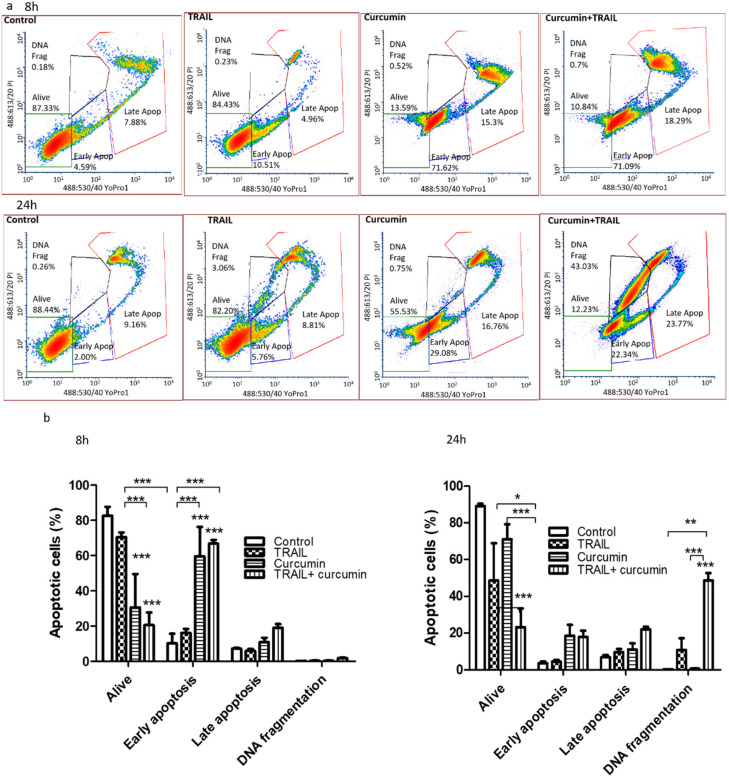
Curcumin or curcumin+TRAIL induced apoptotic cell death. ACHN cells were cultured on six-well plates and exposed to 25 µM curcumin for 4 h. Following this, the cells were incubated with 0.05% DMSO in culture medium or 50 ng/mL TRAIL for 8 or 24 h (**a**). The treated cells were labelled with YO-PRO-1 (100 µM in DMSO) and propidium iodide (10 µg/mL) immediately prior to flow cytometry analysis. (**a**) Representative scatter plots show and compare the effects of the treatments at two different time points (8 and 24 h). (**b**) Results are presented as the mean of the percentage of early apoptosis, late apoptosis, and DNA fragmented cells induced by a specific treatment ± SEM of three independent experiments. *, ** and *** indicate statistically significant difference from respective control at *p* < 0.05, *p* < 0.01, and *p* < 0.001, respectively. Two-way ANOVA was used to analyse the data.

Flow cytometry employing the YO-PRO and PI double staining was conducted to determine the exact mechanism of cell death induced by curcumin, TRAIL or curcumin/TRAIL combination in ACHN cells at an early and late time point, 8 and 24 h (Figure 4). The results indicated that the majority of cells were alive in the untreated and TRAIL treatments (82.62 ± 5.14% and 70.49 ± % respectively). Interestingly, the findings indicated that following 8-h exposures more than half of the cell population underwent early stage apoptosis in response to the curcumin and the curcumin plus TRAIL combination treatment (60 ± 16.2% and 61 ± 3.46%, respectively) (Figure 4a). At the later 24-h time point, a significant population of ACHN cells had proceeded to the late stage of apoptosis, followed by DNA fragmentation (Figure 4b). The curcumin and TRAIL combination treatment was shown to accelerate the apoptosis process, with almost 90% of cells undergoing apoptotic cell death at different stages with 17.90 ± 3.38% in early stage apoptosis, 22.02 ± 1.35% in late stage apoptosis, and 48.59 ± 4.03 as DNA fragmented cells (Figure 4b). Cells also showed the typical and ideal pattern of apoptosis and no necrotic events were recorded.

### 3.4. Curcumin/TRAIL Combination Treatment Induces a Caspase-Dependent Apoptosis

**Figure 5:**
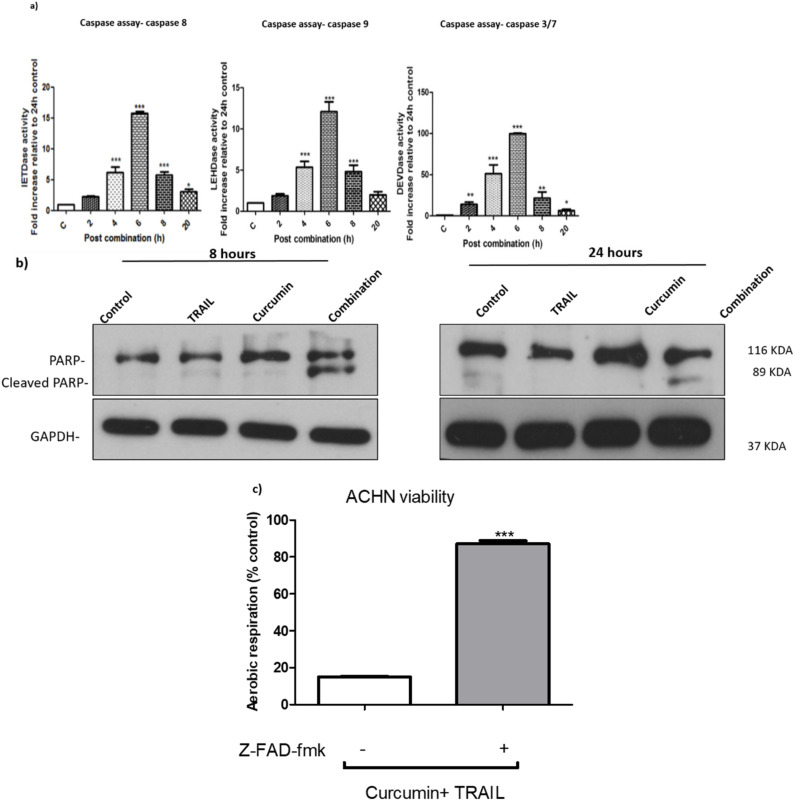
Curcumin/TRAIL combination treatment induced apoptosis via induction of caspases activation (**a**) ACHN cells were cultured in six-well plates for 24 h. The cells were then incubated with culture medium containing 0.5% DMSO or 25 µM curcumin for 4 h, followed by a further incubation vehicle or 50 ng/mL TRAIL for a further 2, 4, 6, 8 and 20 h. Fluorescence was kinetically detected by a scanning fluorescent microplate reader for a period of 120 min (120 cycles, one measurement per minute) at 37 °C at an emission and excitation of 400 and 505 nm, respectively. The results were normalized against protein content and presented as mean ± SEM of five independent experiments. One-way ANOVA was used to analyse the data (**b**) ACHN cells were cultured on six-well plates and treated with culture medium containing 0.5% DMSO, 50 ng/mL TRAIL, 25 µM curcumin or a co-treatment of 50 ng/mL TRAIL/25 μM curcumin. Whole cell protein was extracted at 8 and 24 h with RIPA buffer. Equal amounts of protein were separated by SDS-PAGE electrophoresis, transferred to nitrocellulose and indirectly probed for PARP using monoclonal antibodies. GAPDH was employed as a loading control. A representative blot is shown from three independent experiments. (**c**) ACHN cells were cultured on six-well plates either pre-treated with vehicle or 200 µM z-VAD-fmk for 1 h, then incubated with 25 µM curcumin for 4 h, followed by a further incubation with 50 ng/mL TRAIL for up to 24 h. Cell viability was assessed using FluoroFire-Blue ProViaTox Resazurin Fluorescent assay. *, ** and *** indicate statistically significant differences from control at *p* < 0.05, 0.01, and 0.001, respectively. Independent *t*-test was employed to analyse the data.

To investigate whether caspases were participating in curcumin/TRAIL-induced apoptosis, a caspase assay was carried out to determine the activity of caspase 8, 9, and 3/7. All caspases were shown to be activated following 2-h exposure to the curcumin and TRAIL combination, with a peak in activity levels detected at the 6-h post combination (Figure 5a). The activation of caspase 3/7 was validated by Western blot at 8 and 24 h (Figure 5b). Furthermore, the cleavage of poly (ADP-ribose) polymerase or PARP, a nuclear caspase-3 substrate and a hallmark of apoptosis, was also observed following the exposure to the combination treatment. Caspase-dependent apoptosis was confirmed by pre-treatment of the ACHN cells with the pan caspase inhibitor z-VAD-fmk, which effectively reversed the cytotoxicity of curcumin/TRAIL combination on ACHN cells (Figure 5c).

### 3.5. The Effect of Curcumin, TRAIL, and Curcumin/TRAIL Combination on Pro- and Anti-Apoptotic PROTEIN Expression

**Figure 6:**
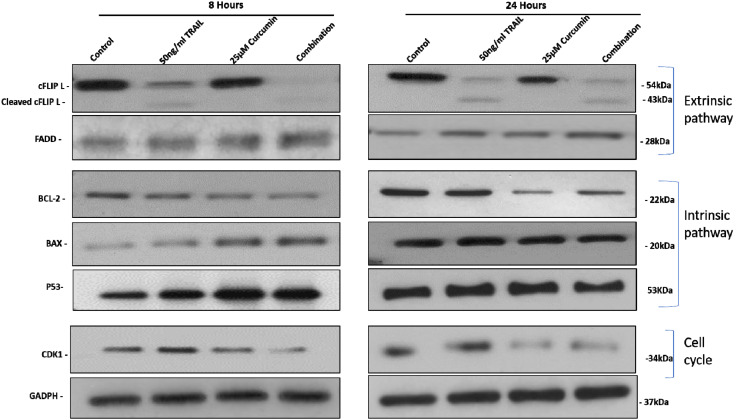
The effect of curcumin, TRAIL, and curcumin/TRAIL combination on the expression of pro-and anti-apoptotic target proteins in ACHN cells. Cells were cultured on six-well plates and treated with vehicle control, 50 ng/mL TRAIL, 25 µM curcumin or 50 ng/mL TRAIL/25 µM curcumin combination treatment. After exposure for 8- and 24-h whole cell lysates were prepared with RIPA buffer. Equal amounts of proteins were separated by SDS-PAGE electrophoresis, transferred to nitrocellulose and indirectly probed for (Bax, BCL-2, P53, FADD, cFLIP and CDK1primary antibodies. GAPDH was employed as a loading control. A representative blot is shown from three independent experiments.

The effects of curcumin, TRAIL and curcumin/TRAIL combination treatments were investigated on the protein expression of several pro/anti-apoptotic genes at early and late time points (8 and 24 h) (Figure 6). Interestingly, curcumin, alone or in a combination with TRAIL, clearly upregulated the expression of Bax FADD and P53 at the earlier time point. However, no induction can be observed at 24 h. An upregulation of FADD can be observed after 24 h treatment with TRAIL, curcumin and both combined. Curcumin, alone or in a combination with TRAIL markedly decreased the anti-apoptotic gene Bcl-2 at 8 h, which can also be observed to a lesser extent at 24 h. cFLIP was shown to be downregulated following treatment with curcumin + TRAIL with a slight downregulation following TRAIL only but not curcumin treatment. Furthermore, curcumin and curcumin/TRAIL combination, but not TRAIL or control, exhibited a marked inhibition of CDK1 at 8 and 24 h treatments.

### 3.6. Curcumin, Alone or in a Combination with TRAIL, Inhibited Proteasome Activity

**Figure 7:**
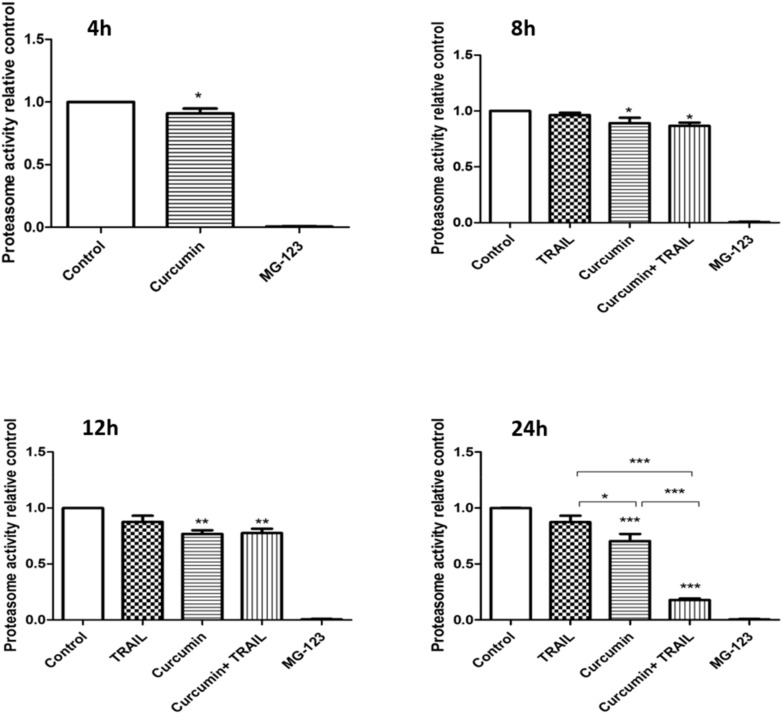
Effect of curcumin, TRAIL, and curcumin + TRAIL combination on proteasome activity in ACHN cells. ACHN cells were cultured on six-well plates incubated with culture medium containing 0.05% DMSO or 25 µM curcumin for 4 h. Following this, cells were either analysed or further incubated with culture medium or 50 ng/mL TRAIL for 4, 8, 12 and 24 h. following the incubation period, cells were broken in lysis buffer. Lysates were incubated for 1 h with 100 µM of SUC-LLVY-AMC fluorogenic substrates specific for 20S subunit of proteasome. The release of (7-amino-4-methyl-coumarin) AMC was fluorometrically measured using an excitation wavelength of 360 nm and an emission wavelength of 475 nm. The assay was run over a period of 60 min (60 cycles, one measurement per min) at 37 °C. To ensure that the observed activity was indeed proteasome derived, 10 µM MG-132 was added to another technical replicates of the vehicle- treated cell lysate, which then served as a positive control for the assay. The results were normalized for protein contents using BCA protein assay and presented as mean ± SEM of three independent experiments. *, ** and *** indicate statistically significant differences relative to the control at *p* < 0.05, 0.01 and 0.001, respectively. One-way ANOVA was used to analyse the data.

The chymotryptic proteasome activity was investigated by measuring the catalytic activity of the 20S subunit of proteasome using a fluorogenic substrate, SUC-LLVY-AMC (Figure 7). When ACHN cells were exposed to 25 µM curcumin only for 4 h, the chymotryptic activity of the 26S proteasome significantly (*p* < 0.05) decreased, and the percentage of inhibition was 9 ± 0.039% compared to the control. Proteasome inhibition interestingly increased with increasing the incubation period to 11 ± 4.9% and 13.35 ± 2.93% at 8 h, 23.14 ± 3.14%, and 22.49 ± 3.84% at 12 h, and 29.58 ± 5.25% and 82.21 ± 1.38% at 24 h for both curcumin alone and curcumin plus TRAIL treatments, respectively. No inhibition in proteasome activity was recorded following the exposure to TRAIL only treatment compared to the control treatment. MG-123 was employed as a positive control for proteasome inhibition.

### 3.7. Curcumin/TRAIL Combination Treatment Induced ROS Induction

**Figure 8:**
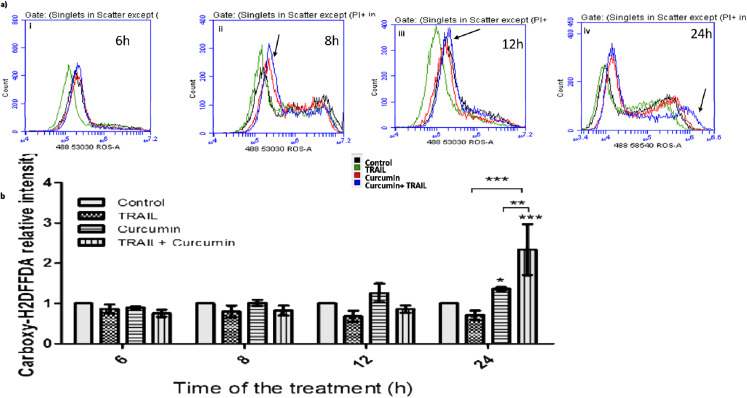
ROS induction by curcumin/TRAIL combination. (**a**) ACHN cells were cultured on six-well plates and treated with vehicle control or 25 μM curcumin for 4 h, followed by a further incubation with 50 ng/mL TRAIL for an additional 2, 4, 8 and 20 h. Therefore, the final exposure length was (**i**) 6, (**ii**) 8, (**iii**) 12 and (**iv**) 24 h. ROS was measured using Carboxy-H2DFFDA probe and analysed by flow cytometry. Negative control cells were incubated with 0.05% DMSO containing complete culture medium only. Arrows indicate a high ROS level following curcumin + TRAIL compared to the other treatments. (**b**) Flow cytometry data was presented as mean relative fluorescent intensity ± SEM of three independent experiments. Two-way ANOVA was used to analyse the data.

Curcumin and curcumin/TRAIL combination treatments were shown to increase ROS level in ACHN cells after only 2 h of the exposure to the combination treatments (Figure 8a i). The increase in ROS level increased after 4 and 8 h of the exposure to the curcumin/TRAIL combination (Figure 8a ii,iii) with the highest observed level at the latest measured time point (24h) (Figure 8a iv). A measurably significant ROS induction was recorded at 24 h post-exposure to both curcumin and the curcumin/TRAIL combination (Figure 8b). Therefore, the 24-h time point was selected to further investigate the effect of curcumin (or curcumin/TRAIL combination)-induced ROS induction on downstream targets, including death receptors (DRs), the CCAAT-enhancer-binding protein homologous protein (CHOP) and MAPKs.

### 3.8. Curcumin/TRAIL Combination Treatment Induced DR4 Upregulation

**Figure 9:**
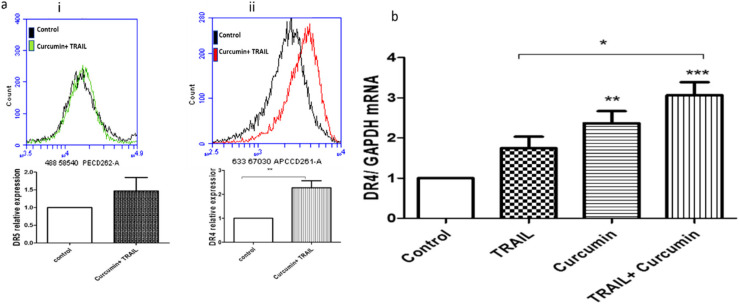
Death receptor 4 upregulation by curcumin/TRAIL combination. (**a**) ACHN cells were cultured on six-well plates and treated with vehicle control or 25 μM curcumin for 4 h, followed by a further incubation with 50 ng/mL TRAIL for up to 24 h. Cells were incubated with (**i**) APC- or (**ii**) PE-conjugated monoclonal anti-human DR4 and DR5 antibodies, respectively. Independent t-test was used to analyse the data. (**b**) Following cell treatment, RNA was isolated and cDNA was synthesized. qRT-PCR was then performed using a TaqMan-based primer specific to DR4. Results were normalized to GAPDH. The figure is a representative of 3 independent experiments. One-way ANOVA was used to analyse the data.

Flow cytometric analysis demonstrated that the surface expression of DR4 and DR5 were upregulated following curcumin/TRAIL combination treatment (Figure 9a i,ii). However, only DR4 was shown to be significantly upregulated following the combination treatment in comparison to the control cells. The results were further confirmed by measuring DR4 expression at mRNA level. DR4 gene expression was shown to be significantly upregulated following exposure of ACHN cells to 25 µM curcumin (*p* < 0.01) or 25 μM curcumin/50 ng/mL TRAIL (*p* < 0.001) in comparison to the untreated control. Additionally, curcumin/TRAIL combination has shown a significant (*p* < 0.05) upregulation of DR4 compared to the TRAIL only treatment (Figure 9).

### 3.9. ROS Regulates Curcumin or Curcumin/TRAIL Combination-Induced CHOP Activation and DR4 Upregulation

**Figure 10:**
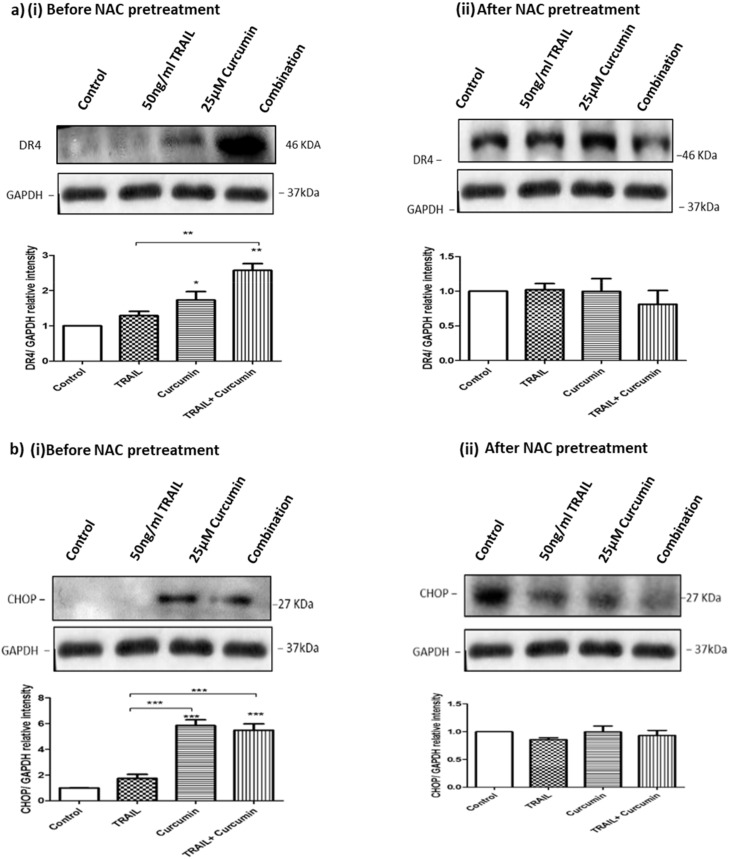
NAC pre-treatment abrogates CHOP and DR4 activation by curcumin or curcumin/TRAIL combination. ACHN cells were pre-treated with 0.5% DMSO-containing culture medium, 50 ng/mL TRAIL, 25 µM curcumin or a co-treatment of 50 ng/mL TRAIL + 25 μM curcumin. The cells were pre-treated with 10 mM N-acetyl cysteine (NAC) for 1 h to block ROS activity prior to exposure to the various treatments for 24 h. Whole cell protein was extracted at 24 h with RIPA buffer. Equal amounts of protein were separated by SDS-PAGE electrophoresis, transferred to nitrocellulose and indirectly probed for (**a**) DR4 and (**b**) CHOP using monoclonal antibodies and an ECL detection system. GAPDH was used as a loading control. *, ** and *** indicate statistically significant differences from respective controls at *p* < 0.05, *p* < 0.01 and *p* < 0.01. One-way ANOVA was used to analyse the data.

To investigate whether ROS could affect DR4 expression, ACHN cells were pre-treated with 10 mM of N-acetylcysteine (NAC), an artificial antioxidant, for 1 h prior to exposure to the other treatments for up to 24 h. Exposure of the ACHN cancer cells to curcumin or the curcumin/TRAIL combination markedly upregulated DR4 protein expression compared to TRAIL alone or the control cells. However, NAC pre-treatment of the ACHN cells effectively suppressed the effect of curcumin or curcumin/TRAIL combination on DR4 protein expression (Figure 10a i,ii). In addition, the effects of the treatments on CHOP were assessed, with both curcumin and the curcumin/TRAIL combination treatment resulting in a significant increase in CHOP expression in comparison to the TRAIL alone and control treatments. Pre-treatment with NAC resulted in similar expression levels across all treatments in comparison to the controls (Figure 10b i,ii).

### 3.10. ROS Regulates Curcumin or Curcumin/TRAIL Combination-Induced MAPK Dysregulation

**Figure 11:**
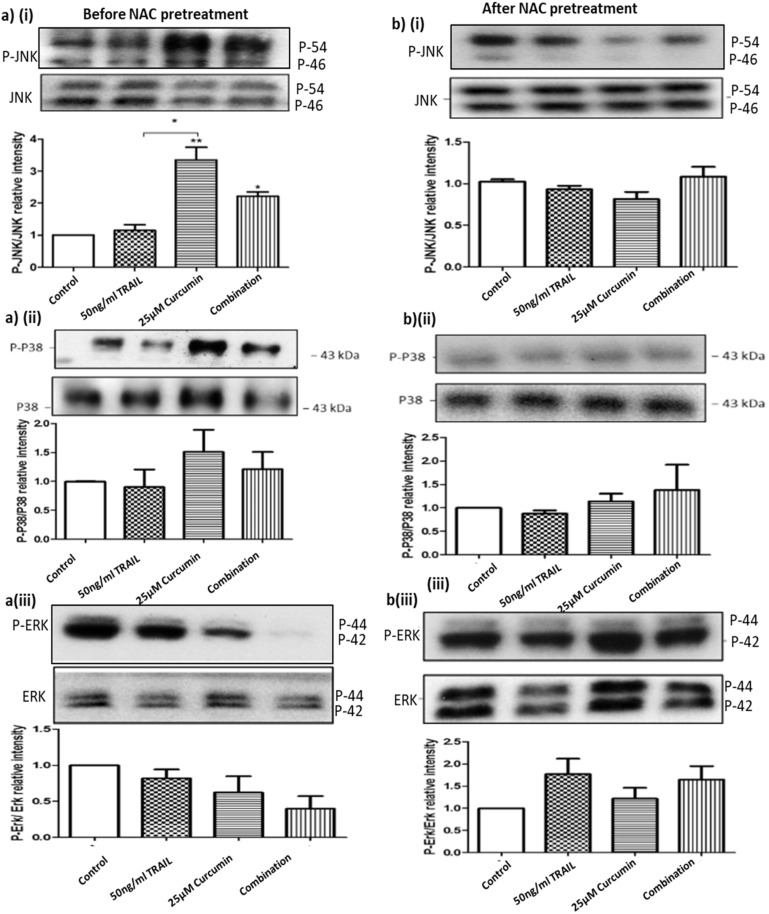
NAC pre-treatment abolished curcumin or curcumin/TRAIL combination-induced MAPK dysregulation ACHN cells were pre-treated with control medium (0.05% DMSO), 50 ng/mL TRAIL, 25 µM curcumin or 50 ng/mL TRAIL/25 μM curcumin combination treatment for further 24 h. Whole cell lysates were prepared in RIPA buffer. Equal amounts of protein were separated by SDS-PAGE electrophoresis, transferred to nitrocellulose and indirectly probed for p-JNK, JNK p-P38, P38, and p-ERK and ERK using monoclonal antibodies and ECL detection system. To assess the effect of pre-treatment with an antioxidant the ACHN cells were incubated with (**bi–iii**) or without (**ai–iii**) 10 mM NAC for 1 h prior to treatment of the cells as previously described. Whole cells lysates were assessed by Western blot indirectly probing for the aforementioned primary antibodies. One-way ANOVA was used to analyse the data.

Previous studies have implicated MAPK signalling pathways in renal cellular damage, therefore their role in ROS dysregulation was also examined. Treatment with curcumin or curcumin/TRAIL combination was shown to affect the pro-apoptotic and stress regulating MAPK signalling proteins, JNK and p38, as well as the pro-survival protein ERK (Figure 11). Analysis of basal JNK expression showed its expression was unchanged regardless of treatment. While the protein level of the phosphorylated protein (P-JNK) was significantly elevated following the exposures to curcumin (*p* < 0.01 and curcumin/TRAIL combination (*p* < 0.05) (Figure 11a i). Despite the increase of P-P38 level after targeting the cells with curcumin or curcumin/TRAIL combination, the increase in P-38 activation following the treatments was not significant (Figure 10a ii). On the other hand, targeting cells with aforementioned treatments clearly reduced the level of P-ERK with no effects on basal ERK level (Figure 11a iii). Pre-treating cells for 1 h prior to the onset of treatments reversed the potential dysregulating effects of curcumin or curcumin/TRAIL treatments on JNK, P-38 and ERK. However, no effects were seen on the basal levels of those proteins (Figure 11b i–iii).

### 3.11. JNK Regulated CHOP, but Not DR4 Activation

**Figure 12:**
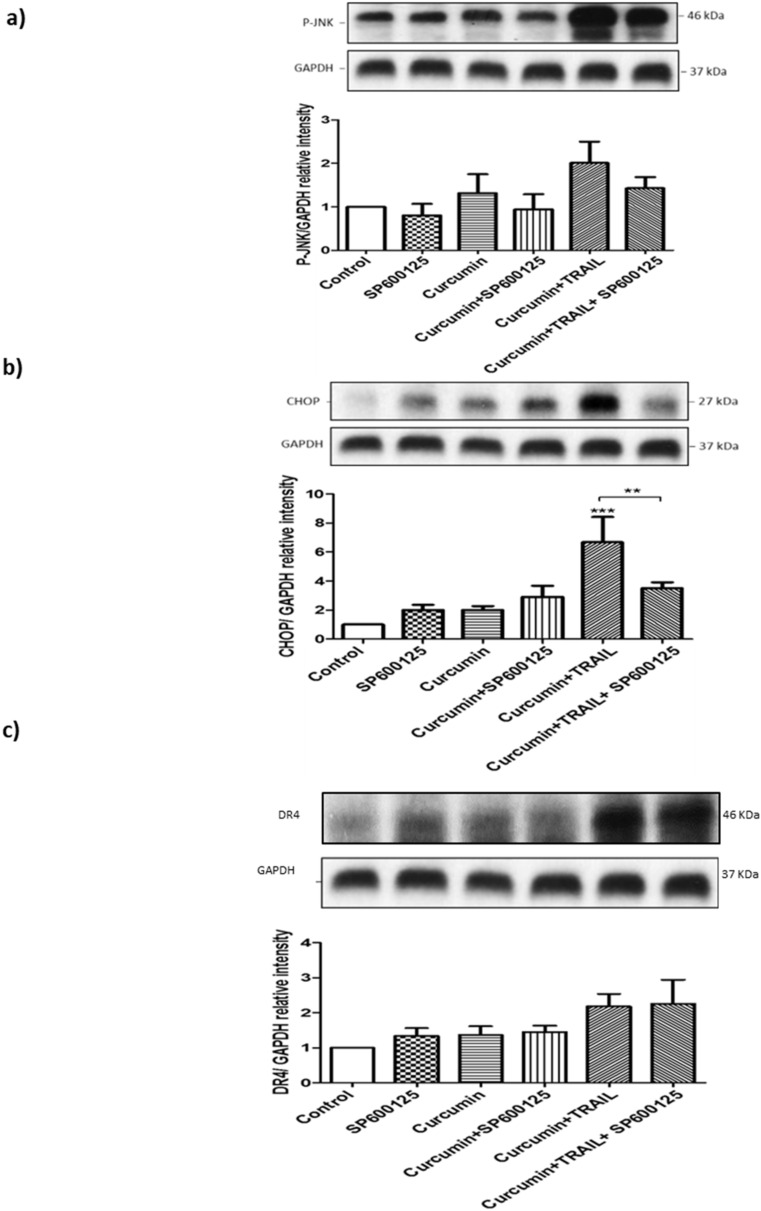
CHOP, but not DR4, was activated by JNK following curcumin/TRAIL combination treatment ACHN cells were either pre-treated with DMSO or 10 μM JNK inhibitor (SP600125) for 1 h. Cells were then treated with vehicle control, 50 ng/mL TRAIL, 25 µM curcumin or 50 ng TRAIL/25 μM curcumin. Whole cell protein was extracted at 24 h with RIPA buffer. Equal amounts of protein were separated by SDS-PAGE electrophoresis, transferred to nitrocellulose and indirectly probed for (**a**) P-JNK, (**b**) CHOP and (**c**) DR4 using a mAb and ECL detection system. GAPDH was used as a loading control. Representative blots are shown from 3 independent experiments. ** = *p* < 0.01 and *** = *p* < 0.001. One-way ANOVA was used to analyse the data.

To address whether ROS upregulated CHOP and DR4 expressions via JNK, cells were pre-treated with 10 µM SP600125, a JNK II inhibitor, before exposure to 50 ng/mL TRAIL, 25 μM curcumin or the 25 μM curcumin/50 ng/mL TRAIL combination (Figure 12). Pre-treating cells with SP600125 significantly inhibited CHOP upregulation following the combination treatment. However, JNK blockage did not affect DR4 upregulation induced by the indicated combination (Figure 12).

### 3.12. Assessment of Treatments Toxicities and Effectiveness Using Zebrafish In Vivo Testing Model

**Figure 13:**
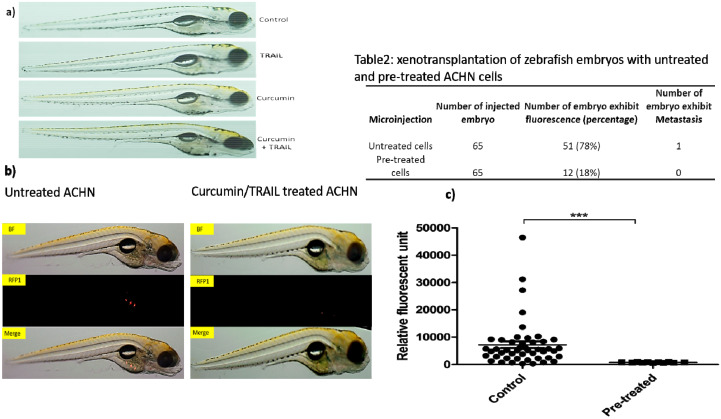
In vivo testing of the treatments using zebrafish embryos. Adult wild fish were set in breeding tanks. Next day, the eggs were collected, washed, inspected and placed in embryo Danieau’s medium. (**a**) 48 h post-fertilization (48 hpf), at least, 10 embryos were placed in each well of a 48-well plate containing 400 µL embryo medium. The treatments, which included 25 µM curcumin, 50 ng/mL TRAIL or 25 µM curcumin + 50 ng/mL TRAIL, were added to the embryonic medium for 72 h. Post-treatment, the fish were anesthetized using 0.002% tricain and inspected under a microscope for any abnormalities or death. Representative light microscopy images were taken using an Olympus SZX10 microscope. (**b**) At least 25 larvae were injected with the ACHN cells labelled with vibrant Dil (red) in their yolk sac using a SYS-PV830 microinjector system. At 72 h post-injection, the larvae were anesthetized with 0.002% tricain then placed on a glass slide and imaged using a fluorescent Olympus SZX10 microscope. BF: bright field channel, RFP1: red channel, Scale bar is 2 mm, n = 3. (**c**) Statistical analysis using independent t-test to compare between the fluorescent intensity of embryos injected with untreated and pre-treated cells. N = 3 and *** indicates a significance level at *p* < 0.001.

In order to assess the toxicities of the treatments in vivo, two-day old zebrafish embryos were incubated with 25 µM curcumin, 50 ng/mL TRAIL and 25 µM curcumin/50 ng/mL TRAIL combination for 72 h. Following the incubation period, the embryos were inspected. No death or developmental abnormalities were apparent following the exposure of zebrafish embryos to the indicated treatments for 72 h (Figure 13a). Pre-treatment of cells with curcumin/TRAIL combination prior to the injection blocked their ability to form tumour colonies in the engrafted zebrafish embryos compared to the untreated injected cells. Tumour masses were detected in more than 78% of injected embryos with ACHN cells, while small tumour masses could be seen in only 18% of injected embryos with curcumin plus TRAIL-treated ACHN cells (n = 65) which is measurably significant. Curcumin/TRAIL combination-pre-treated ACHN cells exhibited a significant reduction of tumour masses than vehicle-pre-treated ACHN cells. While the results are preliminary, they do confirm the in vitro findings and also recommend the use of a zebrafish model for preliminary metastatic potential analysis (Figure 13b).

## 4. Discussion

The development of resistance to anti-cancer therapies such as TRAIL diminishes any drug’s potential clinical applications as an anti-cancer therapy. Recently, several studies have focused on combining TRAIL with other therapeutics, such as curcumin, in an effort to reduce resistance and improve anticancer activity. We recently reported that curcumin could work as a tumour blocker and suppressor to inhibit carcinogenesis induced by the food additive KBrO_3_ [22]. In this project, we aimed to assess the chemosensitisation potential of curcumin on TRAIL’s resistant kidney cancerous ACHN cells and to provide dynamic insights into the molecular mechanisms of cell sensitization.

Curcumin was shown to augment TRAIL-induced cell death in the ACHN cells, as assessed by the resazurin assay, with the highest level of synergy at 25 uM curcumin + 50 ng/L TRAIL based on the combination index analysis [27,28]. Morphological analysis revealed that the combination of curcumin with TRAIL clearly induced apoptosis with marked apoptotic characteristic features while no toxicity was observed on the normal kidney cells. The uptake and accumulation of curcumin and, thus toxicity was significantly higher in cancerous cells than adherent cells [29]. Following the early exposure to curcumin, more than 85% of cells were detected undergoing apoptosis with more than 70% in the early apoptosis stage. However, these percentages were then reduced to 45% total cell death, in which 29% underwent early apoptosis. Such “transient and reversible” pro-apoptotic effects of curcumin have been well described by previous studies [30,31] indicated that curcumin imposes a remarkable stress on cells, particularly, cell membranes causing perturbation of the membranes leading to influx of the small dye molecules.. Therefore, the population of cells at early apoptosis, dramatically increased at 8 h followed by a reduction at 24 h due to cell membrane re-stabilization while those undergoing late apoptosis and DNA fragmentations did not change. A previous study has reported that the fast action of curcumin is, in fact, due to its lipophilic nature which results in fast incorporation into cell membranes [32]. A number of other studies demonstrated that the phenolic groups of curcumin interact with membrane proteins forming hydrogen bonds upon entering cells, resulting in a disturbance of the cell membrane which increases permeability [30,33,34,35,36,37,38,39,40,41]. This might explain the perturbating effects of curcumin on the cancerous ACHN cell membrane that resulted in sensitising ACHN cells to TRAIL’s pro-apoptotic effect.

We have also shown that curcumin, alone or in a combination with TRAIL, dysregulated several apoptosis-regulating genes. For instance, cyclin-dependent kinase 1, CDK1, an archetypical kinase that plays a central role in cell division through G2 and mitosis phases [42] was shown to be dysregulated. This finding is in agreement with other studies [43,44,45] which suggests that curcumin might sensitise the cells to TRAIL via interfering with ACHN cell cycle progression at G2/M phase. Curcumin and curcumin/TRAIL combination upregulated P53 and Bax while downregulating Bcl2. The dysregulation of the key proteins that regulate the mitochondrial pathway was congruent with flow cytometry findings in which a high population of cells shifted to early apoptosis at 8 h followed by a drop of cell percentage to about 29% at 24 h. Such findings might suggest that, in addition to affecting the plasma membrane, curcumin can perturb mitochondrial membranes causing a “transient “activation of the intrinsic or mitochondrial pathway. Our hypothesis is supported by other studies that have shown that the lipophilic nature of the curcumin molecule facilitates the ease of distribution within the lipophilic components of the membranous structures, such as the ER, mitochondria and nuclear envelopes. The existence of curcumin in these organelles negatively affects their structure and function. In the mitochondria, curcumin has been found to interfere with ATP production triggering increased ROS generation [30,33,34,35,36,37,38,39,40,41].

The effect of curcumin/TRAIL combination was clear on three important extrinsic pathway regulator proteins, DR4, cFLIP and FADD. While the combination inhibited cFLIP, it upregulated FADD. Although curcumin did not show any effect on cFLIP expression, it potentiated the downregulation of cFLIP when combined with TRAIL. Taken all together, curcumin and TRAIL induced apoptotic cell death mainly via activation of the extrinsic, and partly the intrinsic pathways of apoptosis.

Chymotrypsin-like proteasome activity was also examined in this study. The exclusive determination of chymotrypsin-like activity was based on a study by Milacic et al., which has shown that curcumin exhibited a higher activity against the chymotrypsin-like rather than trypsin- or caspase-like activity of the proteasomes [46]. Moreover, previous studies reported that chymotrypsin-like activity of proteasomes is considered the most important one for protein degradation, and a highly attractive target for anticancer drugs. The proteasome plays an integral role in carcinogenesis [47] and several reports have demonstrated that targeting the proteasome can inhibit cancerous cell growth and induce apoptotic cell death in vitro and in vivo [41,48]. Our findings demonstrated the potential activity of curcumin to inhibit proteasome activity, and the maximal inhibition was noticed following the combination with TRAIL. However, TRAIL, by itself, failed to inhibit the chymotryptic 20S subunit of the proteasome. Our results are consistent with previous studies which have shown curcumin can efficiently reduce the chymotryptic activity of proteasome of different cancer cells in vitro and in vivo [46,49,50,51,52,53].

Evidence was also provided to show that curcumin, alone or as part of the combination treatment, induced oxidative signalling in ACHN cells, which was reflected by increased ROS production. These results agree with other studies which highlights curcumin’s pro-oxidant potential as a pivotal mechanism to inhibit cancer cells via upregulation of proapoptotic or downregulation of anti-apoptotic genes [54,55,56,57,58,59]. Several studies have explained curcumin’s pro-oxidant effects. Structurally, due to the α, β-unsaturated β-diketo moiety, curcumin acts as an electrophile which undergoes a nucleophilic addition reaction with anions that act as a nucleophile [60]. Another proposed mechanism is due to the interaction of curcumin with GSH and formation of a curcumin-glutathione complex that can be associated with the intracellular depletion of GSH levels and, therefore, cells become vulnerable to oxidative damage [61,62]. Curcumin has also been shown to target and inactivate redox-equilibrium maintaining enzymes such as thioredoxin reductase enzyme [63].

We also hypothesized that curcumin affected the expression of TRAIL-receptors. To address this question, the surface expression of the death receptors DR4 and DR5, which are the initial triggers of the extrinsic apoptotic pathway, were assessed. A large but non-significant increase in the expression of DR5 was observed in response to curcumin/TRAIL combination. However, the surface expression of DR4 was shown to be significantly upregulated following the exposure to the combination treatment. Recently, it has been shown that activation of DR5 was linked to TRAIL-induced cancer cell migration and invasion and thus drives TRAIL-induced drug resistance. On the other hand, DR4 plays an integral role in activating apoptosis machinery without triggering tumour migration [64], this could shed light on the importance of the higher affinity of curcumin in activating DR4 rather than DR5, thus reducing the likelihood of developing TRAIL-induced cancer cell invasion and drug resistance in renal cancer cells.

Clearly, pre-treatment of the ACHN renal carcinoma cells with the ROS scavenger, NAC, abrogated curcumin or curcumin/TRAIL- induced DR4 activation in our study. This suggests a key role of oxidative stress in TRAIL sensitisation via upregulation of death receptors. This finding is in line with other studies [65,66,67]. To add credence to the idea that increased ROS production promotes apoptosis, the level of CHOP, an endoplasmic reticulum-linked stress protein, was shown to be significantly increased in the curcumin or combination treatments. Blockade of ROS induction through the use of the ROS scavenger NAC was shown to decrease this ER stress, potentially due to curcumin forming adducts with the thiol groups of NAC instead of the ER proteins, thus resulting in decreased expression of CHOP [68].

The action of ROS in apoptosis was also shown to extend beyond the initiation of the pathway, to the MAPK signalling pathways, JNK, ERK and p38, which were found to be ROS-dependent. Both the JNK and p38 MAPK signalling pathways demonstrated activation in response to curcumin alone or the combination treatment, while the expression of P-ERK was shown to be decreased in response to the same treatments. The relationship between CHOP induction and JNK activation has previously been established [69,70,71,72]. These studies hypothesized that ROS activation of the MAPK signalling pathways, in particular JNK activation, is essential for DR4 upregulation and the subsequent activation of CHOP. To assess the validity of this hypothesis, ACHN cells were pre-treated with the JNK inhibitor, SP600124, and the expression of both CHOP and DR4 genes were investigated. Blockage of JNK activation was shown to significantly decrease CHOP expression in response to the combination treatment. These findings are in parallel with other studies [69,73,74].

Finally, the effectiveness of curcumin and the combination of curcumin/ TRAIL treatment as anticancer therapies were assessed in vivo in a zebrafish xenograft model. Within the past decade, zebrafish have dramatically developed to become a powerful model of cancer progression in vivo, with great promise to explore more about human diseases, particularly cancer formation, metastasis and treatment [75]. The striking similarities in molecular and histopathological features between zebrafish and human malignancies, with a genetic homology of about 80%, can allow the further exploration of the underlying mechanisms of tumour formation, development and progression in humans [76]. All treatments, curcumin alone, TRAIL alone and the combination treatment were shown to be safe, not causing lethality or developmental abnormalities in the zebrafish embryos. Interestingly, following xenograft of ACHN cells pre-treated with the combination treatment, a markedly decreased ability to form tumour masses in the embryos was observed in comparison to the embryos injected with untreated ACHN cells. To our knowledge, this is the first study to evaluate the effectiveness of curcumin/TRAIL combination treatments against renal xenografted tumours in zebrafish-based tumour xenotransplantation studies.

## 5. Conclusions

In conclusion, this study shows that the combination of curcumin and TRAIL synergistically inhibited the cancerous ACHN cell growth. Furthermore, curcumin sensitised the cells to TRAIL induced caspase-dependent apoptotic cell death via different mechanisms. Indeed, induction of ROS, activation of the extrinsic and intrinsic pathways of apoptosis, induction of cell cycle arrest, upregulation of death receptors and inhibition of proteasome system were identified to be the main mechanisms. Our study has also shed light on the involvement of ROS as the key player of curcumin sensitization to TRAIL via upregulation of TRAIL-R1, CHOP, and dysregulation of MAPK. Furthermore, our study unequivocally unravelled the involvement of JNK as a regulator for CHOP but not DR4 activation induced by ROS. Thus, ROS/JNK/CHOP and ROS/DR4 pathways are the potential pathways of curcumin sensitisation of ACHN cells to TRAIL. Furthermore, the zebrafish xenotransplantation model revealed promising anti-tumour potential of curcumin/TRAIL combination treatments, which opens up the possibility for rodent-based in vivo studies, as well as human clinical trials to find cheaper, safer, more available and, most importantly, effective anti-cancer therapies against RCC.
